# An asymmetry in past and future mental time travel following vmPFC damage

**DOI:** 10.1093/scan/nsaa163

**Published:** 2020-12-31

**Authors:** Elisa Ciaramelli, Filomena Anelli, Francesca Frassinetti

**Affiliations:** Department of Psychology, University of Bologna, Bologna 40126, Italy; Center for Studies and Research of Cognitive Neuroscience, Department of Psychology, University of Bologna, Cesena 47521, Italy; Department of Psychology, University of Bologna, Bologna 40126, Italy; Department of Surgical and Medical Sciences, “Magna Graecia” University of Catanzaro, Catanzaro 88100, Italy; Department of Psychology, University of Bologna, Bologna 40126, Italy; Istituti Clinici Scientifici Maugeri IRCCS, Operative Unit for Recovery and Functional Rehabilitation, Institute of Castel Goffredo, Mantova 46042, Italy

**Keywords:** mental time travel, self-projection, episodic memory, future thinking, vmPFC

## Abstract

The role of ventromedial prefrontal cortex (vmPFC) in mental time travel toward the past and the future is debated. Here, patients with focal lesions to the vmPFC and brain-damaged and healthy controls mentally projected themselves to a past, present or future moment of subjective time (self-projection) and classified a series of events as past or future relative to the adopted temporal self-location (self-reference). We found that vmPFC patients were selectively impaired in projecting themselves to the future and in recognizing relative-future events. These findings indicate that vmPFC damage hinders the mental processing of and movement toward future events, pointing to a prominent, multifaceted role of vmPFC in future-oriented mental time travel.

## Introduction

Mental time travel (MTT) is the ability to project oneself in subjective time to remember past events and imagine future events (Atance and O’Neill, 2001; Buckner and Carroll, 2007; Suddendorf and Corballis, 2007; Schacter *et al.*, 2012). Past and future MTT are associated with activity in a ‘core network’ of largely overlapping brain regions, including the medial temporal lobes, ventromedial prefrontal cortex (vmPFC), posterior cingulate cortex and posterior parietal cortex bilaterally (Addis *et al.*, 2007; for a review, Schacter *et al.*, 2012). The neural overlap between past and future MTT has been attributed to shared component processes. These include general constructive processes needed to simulate any complex event, whether located in time or atemporal, such as the ability to recover and integrate the individual elements comprising the event (Schacter *et al.*, 2012) in a coherent spatial context (Hassabis *et al.*, 2007). Other processes are temporal in nature, such as the awareness of subjective time (Nyberg *et al.*, 2010) and of one’s protracted existence in subjective time, extending from the personal past, through the present, to the personal future (‘autonoetic awareness’; Wheeler *et al.*, 1997; Tulving, 2002; see Dafni-Merom and Arzy, 2020 for a review).

Despite many commonalities, past and future MTT also differ in obvious ways, which is reflected in partially diverging neural substrates. The future has not happened; it is supposedly constructed assembling elements from multiple episodes and general knowledge, loading heavily on executive functions and semantic knowledge (Weiler at al., 2011; Irish and Piguet, 2013). Coherently, future compared to past MTT is associated with increased engagement of the dorsolateral prefrontal cortex and the lateral temporal lobe (Addis *et al.*, 2007; Schacter *et al.*, 2012). Individuals think about (Baird *et al.*, 2011; Stawarczyk  *et al.*, 2013) and value (Caruso *et al.*, 2008) the future more than the past, which is likely related to the fact that the future can still be shaped by their decisions and is connected to their goals (D’Argembeau and Van der Linden, 2004; D’Argembeau and Mathy, 2011). The frontopolar cortex, an area strongly associated with processing of intentions (Okuda *et al.*, 2003) and personal goals (D’Argembeau *et al.*, 2010), is indeed more active for future than past MTT.

An important question is whether brain regions implicated in MTT contribute necessarily and differentially to future and past MTT. Neuropsychological studies of brain-damaged individuals have shown that lesions to the medial temporal lobes (Hassabis *et al.*, 2007; Rosenbaum *et al.*, 2009; Race *et al.*, 2011; Romero and Moscovitch, 2012) and vmPFC (Bertossi *et al.*, 2016a,b, 2017) impair both past and future MTT, as well as construction of atemporal events, suggesting a role in core constructive processes supporting both past and future MTT. Differently, lesion or dysfunction of the lateral prefrontal (Berryhill *et al.*, 2010; de Vito *et al.*, 2012) and temporal cortex (Irish *et al.*, 2012; Duval *et al.*, 2012) impair future but not past MTT, suggesting a more pronounced role in MTT toward the future.

In this study, we investigate further the role of vmPFC in past *vs* future MTT using a novel approach. Although our previous studies point to a pervasive MTT impairment in vmPFC patients (Bertossi *et al.*, 2016a,b), we have reasons to hypothesize that vmPFC plays a more prominent role in future-oriented MTT. In a previous study, we found that vmPFC (but not control) patients had more difficulties imagining future compared to atemporal events (Bertossi *et al.*, 2016a). Also, vmPFC patients tend not to think about their future during mind-wandering, while they normally experience past- and present-oriented thoughts (Bertossi and Ciaramelli, 2016). In addition, vmPFC patients are long described as ‘myopic’ to the future consequences of their choices (Bechara *et al.*, 1994), and show steep delay discounting of future rewards (Sellitto *et al.*, 2010). We note, however, that vmPFC is also strongly implicated in past MTT. vmPFC damage can indeed result in confabulation, the false recall of events that did not actually happen (Gilboa *et al.*, 2006). Additionally, a recent study found even more pronounced deficits in past compared to future MTT in patients with prefrontal lesions including (though not confined to) vmPFC (Rasmussen and Berntsen, 2018) and one study found no deficit in either (Kurczek *et al.*, 2015). Thus, the effect of vmPFC damage on past and future MTT deserves further inquiry.

We think that methodological aspects of previous studies may have limited our ability to detect differences in future *vs* past MTT in vmPFC patients. First, MTT paradigms typically require participants to verbally report on personal past and future events. These paradigms entail both time consideration and event construction. This is problematic because some of the basic processes underlying event construction appear to be mediated by vmPFC, irrespective of time (Bertossi *et al.*, 2016a; De Luca *et al.*, 2018; McCormick *et al.*, 2018; Lieberman *et al.*, 2019), potentially blurring differences between past and future MTT. In addition, performance depends massively on narrative abilities, and poor narrative abilities would hinder verbal reports of past and future events alike, further contributing to level out past and future MTT performance (Bertossi *et al.*, 2017). Lastly, the richness of constructed experience (e.g. number of details) is a sensitive yet unspecific index of MTT performance. It is the unique output of a multiplicity of processes operating in concert to shape MTT, which might be differentially engaged during past and future MTT, and differentially affected by vmPFC damage. Therefore, comparing and contrasting past and future MTT in vmPFC patients requires a more fine-grained analysis of different component processes of MTT.

This is the novel approach we take to study the role of vmPFC in past and future MTT. Arzy *et al.* (2008) proposed that MTT is supported by two independent components: self-projection, the imagination of the self in different moments of subjective time, and self-reference, the relation between the assumed time perspective and an event (relative-past or relative-future; Arzy *et al.*, 2008; Dafni-Merom and Arzy, 2020; but see Rogers *et al.*, 1977). For example, events from last year become (are experienced as) future if contemplated from 15 years back in the past. To study different MTT components and their neural substrate, we had vmPFC patients and brain-damaged and healthy controls mentally project themselves to a future, past or present point in subjective time (self-projection). They were then presented with a series of events (e.g. first son) that they had to classify as past or future relative to the currently assumed time perspective (self-reference). If vmPFC is prominently involved in future MTT, as we predict, vmPFC patients should be impaired, compared to the control groups, in projecting themselves to future compared to past or present moments of subjective time and in recognizing future compared to past events.

## Materials and methods

### Participants

Participants included 14 patients with brain damage and 16 healthy individuals (see Table 1 for individual patients’ demographic and clinical data). Patients were recruited at the Centre for Studies and Research in Cognitive Neuroscience (Cesena, Italy) and at the Istituti Clinici Scientifici Maugeri IRCCS (Castel Goffredo, Italy), on the basis of their lesion site (see below), as documented by magnetic resonance imaging (MRI) or computerized tomography (CT) scans. Seven patients had lesions centered on vmPFC (vmPFC patients, 4 males, mean age: 60.43 years, s.d. = 9.02; mean years of education: 10.14, s.d. = 2.67). vmPFC patients’ lesions resulted, in all cases, from rupture of an aneurysm of the anterior communicating artery and were bilateral. The remaining seven patients had brain lesions that did not involve vmPFC (control patients, 4 males, mean age: 55.43 years; s.d. = 15.86; mean years of education: 9.6, s.d. = 3.36). Control patients’ lesions were caused by ischemic or hemorrhagic stroke, traumatic brain injury or brain tumor and were in the left hemisphere in two cases and in the right hemisphere in five cases. Lesion sites mainly included the occipital cortex (three cases), the occipito-temporal area (five cases) and the parietal cortex (three cases). For one of the seven control patients, the lesion description was available but MRI scans were not available, and therefore, we could not reconstruct precisely the extension of the lesion. There was no significant difference in lesion volume between vmPFC patients and the remaining six control patients (47 *vs* 30 cc, *t*_11_ = 1.47, *P* = 0.16). Included patients were in the stable phase of recovery (at least 1 year post-morbid). The healthy control group comprised 16 participants without neurological or psychiatric history matched to the patients on mean age and education (7 males, mean age: 60.56 years, s.d. = 4.21; mean years of education: 12.69, s.d. = 2.87).

**Table 1.** Patients’ demographic, clinical and neuropsychological data

	vmPFC patients							Control patients						
	p. 1	p. 2	p. 3	p. 4	p. 5	p. 6	p. 7	p. 1	p. 2	p. 3	p. 4	p. 5	p. 6	p. 7
Sex	M	M	M	F	F	M	F	M	F	M	F	F	M	M
Age (years)	58	58	47	70	74	56	60	56	31	58	57	65	80	41
Education (years)	8	8	13	8	8	13	13	8	13	8	13	5	7	13
Time since lesion (years)	14	8	8	1	15	5	1	1	1	2	1	4	1	1
Raven’s Standard Matrices (cutoff = 15)	33.5	31	32.5	31.5	33.5	27	33.75	27.5	30.25	30	35.5	32.75	30.75	32.25
Phonemic fluency (cutoff = 17)	27	26	21	41	31	32	22	27	22	25	50	30	29	44
Semantic fluency (cutoff = 25)	37	54	40	61	47	35	42	56	43	32	52	42	32	72.7
Stroop test—errors (cutoff = 4.23)	0	0.5	0	0	3.5	0	4.75*	0	0	1.25	0	0.5	1.5	-
Stroop test—interference times (cutoff = 36.91)	17	13.5	26.2	16.5	17	16	16.5	15	22	15.75	14.75	23.5	21.25	-
Short-term memory—digit span (cutoff = 3.75)	5	5	6.5	6.25	5.25	5.75	4.75	5	5.5	6	5.75	6.5	6.25	6.5
Long-term memory—prose passage recall (cutoff = 4.75)	5	10.9	13	4*	5.5	13.5	14.7	16	-	13.75	16	10.5	13	12.75
Bells cancellation test—total omissions (cutoff <5)	0	2	2	0	0	0	0	0	0	0	1	4	4	0
Bells cancellation test—left omissions (cutoff <5)	0	0	0	0	0	0	0	0	0	0	1	1	2	0
Apples test—full apples barrage score barrage (cutoff = 45)	50	50	48	50	50	50	50	50	46	49	50	50	48	50
Apples test—full apples asymmetry score (cutoff = 2)	0	0	2	0	0	0	0	0	0	1	0	0	0	0
Line bisection test—long lines (cutoff = 5.73)	−0.55	0.25	0.63	0.25	−0.33	0.04	0.04	−0.55	0.30	0.40	−0.05	−0.57	−0.88	0.60
Line bisection test—short lines (cutoff = 2.53)	0.00	0.38	−0.50	0.37	0.03	0.30	0.30	0.00	0.23	−0.10	−0.22	0.18	−1.23	0.00

The table reports, for each patient (p), scores corrected for age, education and gender according to normative samples. For each test, we also report the cutoff score. Scores below the cutoff are considered indicative of impaired performance (corresponding to a percentile <5) and signaled by an *. Dashes indicate missing data.

Participants gave written informed consent to participate in the experiment, which was performed in agreement with the 2008 World Medical Association Declaration of Helsinki and approved by the Bioethical Committee of the University of Bologna and the CEIIAV Ethical Committee of Emilia-Romagna Regional Health Service.

### Lesion analysis

**Fig. 1.**
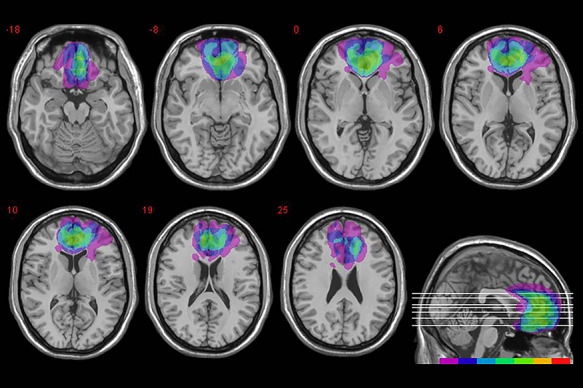
Location and overlap of brain lesions. The panel shows the lesions of the seven patients with vmPFC damage projected on the same seven axial slices and on the mesial view of the standard Montreal Neurological Institute brain. The level of the axial slices is indicated by white horizontal lines on the mesial view of the brain and by *z*-coordinates. The color bar indicates the number of overlapping lesions. Maximal overlap occurs in BAs 11, 10 and 32 of vmPFC. In axial slices, the left hemisphere is on the left side.

Patients’ individual lesions derived from the most recent MRI or CT scans were manually drawn by a trained neuroscientist directly on each slice of the normalized T1-weighted template MRI scan from the Montreal Neurological Institute distributed with MRIcro (Rorden and Brett, 2000). The MRIcro software was used to estimate lesion volumes (in cc) and generate lesion overlap images. Figure 1 shows the extent and overlap of brain lesions in vmPFC patients. Brodmann’s areas (BA) mainly affected were BA 10, BA 11, BA 24, BA 25 and BA 32, though one patient also had damage to BA 46 and BA 47 accounting for about 10% and 18% of lesion size, respectively. The region of maximal lesion overlap occurred in BA 11 (*M* = 17.75 cc, s.d. = 10.68); BA 10 (*M* = 9.79 cc, s.d. = 7.79) and BA 32 (*M* = 7.28 cc, s.d. = 5.60).

### Neuropsychological assessment

A standardized neuropsychological assessment showed that patients’ general cognitive functioning was generally preserved, as indicated by the scores obtained in the Raven’s Standard Matrices, which were within the normal range in all cases and similar between patient groups (*z* = 0.83, *P* = 0.46) (see Table 1 for individual patients’ neuropsychological data). Patients generally reported normal scores in verbal short-term memory also, as indexed by the digit span test (*z* = −1.23, *P* = 0.26; Spinnler and Tognoni, 1987), and in long-term memory, as assessed in a prose passage recall task (*z* = −1.50, *P* = 0.14; Spinnler and Tognoni, 1987), with the exception of a vmPFC patient who showed impaired recall (Table 1). Patients exhibited normal performance in tests assessing executive functions, such as phonemic (*z* = −0.57, *P* = 0.62) and semantic fluency (*z* = −0.13, *P* = 0.90), and, on average, in the Stroop test (number of errors: *z* = 0.07, *P* = 0.94; interference times: *z* = −1.43, *P* = 0.25; Spinnler and Tognoni, 1987), with the exception of a vmPFC patient who showed a pathological number of errors (Table 1). Visuo-spatial abilities were within the normal limits in all cases, as assessed through the Bells cancellation test (total number of omissions: *z* = −0.57, *P* = 0.62; number of left omissions: *z* = −1.34, *P* = 0.20; Gauthier *et al.*, 1989); the line bisection test (long lines: *z* = 0.51, *P* = 0.62; short lines: *z* = 1.73, *P* = 0.10) and the apples test (number of full apples barrage: *z* = 0.89, *P* = 0.38; full apples asymmetry score: *z* = 0.7, *P* = 0.91) (Mancuso *et al.*, 2015).

### Mental time travel task

**Fig. 2.**
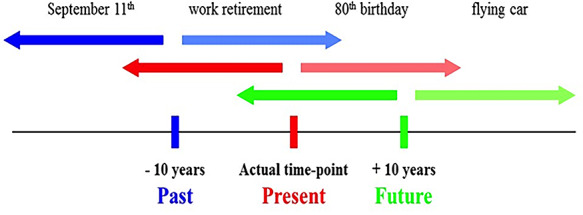
Stimuli and procedure. Participants were required to project themselves in three different locations of subjective time (past, present or future) (self-projection), and to determine whether events were past or future relative to the currently assumed location in subjective time (self-reference).

Participants sat in front of a 15-inch color monitor, at a distance of about 60 cm. In each trial, participants listened to a brief description of an event. Both personal (e.g. car license and first child) and non-personal world events (e.g. Obama’s election and Chernobyl disaster) were included for comparison purposes (see also Anelli *et al.*, 2016a, and Supplementary Table for the complete list of items). The task was to indicate, for each event, whether it had already happened (‘relative past event’) or was yet to happen (‘relative future event’). There were three different conditions, which corresponded to three different locations in subjective time (Figure 2). In the ‘present self-projection condition’, participants answered the questions while imagining themselves as located in the present time; in the ‘past self-projection condition’, they answered the questions while imagining themselves as located 10 years back in the past and in the ‘future self-projection condition’, they answered the questions while imagining themselves as located 10 years ahead in the future. Thus, in each self-projection condition, participants had to determine whether the event being presented was located in the past or the future relative to the currently assumed location in subjective time. The present self-projection condition was always run first, because a pilot study revealed that this made the task more easily comprehended by older adults (Anelli *et al.*, 2016a), and therefore we deemed this procedure suited for brain-damaged individuals. The past and future self-projection conditions were run second or third (counterbalanced).

Each self-projection condition included 24 stimuli, half personal and half non-personal, equally distributed between relative-past and relative-future events, for a total of 72 trials, presented in random order. Events were similar to those employed in the fMRI (functional magnetic resonance imaging) study by Arzy *et al.* (2008), though adapted to the Italian population, and have already been used in a previous work from our laboratory (Anelli *et al.*, 2016a). The same personal and non-personal events were presented to all participants (as in Arzy *et al.*, 2008; Anelli *et al.*, 2016a; see Supplementary Table for the complete list of events). Personal events referred to events that normally characterize important stages of one’s personal life (e.g. birth of a child and car license), whereas non-personal events referred to world-famous, publicly known events (e.g. Obama’s election and Chernobyl disaster). Events were sampled by trying to broadly equate the time distance of relative-past and relative-future events from the assumed time perspective across self-projection conditions. For personal events, across self-projection conditions, participants evaluated relative-past events likely to have taken place up to 30/40 years before the assumed time perspective (e.g. in the past self-projection condition: the first day of school) and relative-future events likely to take place in up to 30/40 years (e.g. for the past self-projection condition: child retirement). Clearly, personal events were selected and attributed to the different time categories based on culturally shared knowledge of what typically happens and when within an idealized life course. People‘s lives, however, deviate from life scripts (whether more or less). At the end of the experiment, therefore, individual participants were interviewed to make sure that personal events were indeed relevant to their own life story, and that the attribution of events to the relative-past/future categories was correct with respect to its chronology (see below). As for non-personal world events, relative-past events generally required, again, to travel back in time up to 30/40 years before the assumed time perspective (e.g. for the past self-projection condition: man on the moon). Relative-future non-personal events were in most cases hypothetical, and therefore not easy to locate in a precise future decade, although most of them were likely to belong to the far future (e.g. for the past self-projection condition: completely defeat mafia).

Because events sampled from different lifetime periods may have different salience and status in memory (e.g. in the past self-projection condition some events belonged to time periods associated with the ‘reminiscence bump’; see Rubin *et al.*, 1998), in a previous study using the same material (Anelli *et al.*, 2016a), an independent group of healthy adults with similar age to those in the present study rated all events on a Likert scale for importance (from 1 = ‘event not important at all’ to 5 = ‘very important/life changing event’) and emotion (from 1 = ‘event eliciting low levels of emotion’ to 5 = ‘event eliciting high levels of emotion’). We found that relative-past and relative-future events presented in different (past, present and future) self-projection conditions did not differ in importance or emotion. Personal (compared to non-personal) events were associated with higher levels of emotion, but comparable importance (Anelli *et al.*, 2016a).

Each trial started with the appearance of a cross in the center of the computer screen for 1000 ms, followed by a black screen and the acoustic presentation of the event through headphones. Across self-projection conditions, participants had to respond whether the event had already happened (‘relative past event’) or was yet to happen (‘relative future event’), by pressing one of two computer keys using the index and middle finger of their right hand (counterbalanced). There was no time limit for responding. At the beginning of the task, participants received written instructions on the computer screen, which in part varied depending on the self-projection condition. In the past/present/future self-projection conditions, they read: ‘Imagine being in the past, 10 years ago/Imagine being in the present/Imagine being in the future, 10 years from now’. Across conditions, they then read: ‘You will be presented with a series of events. Respond with the index/middle finger if the event has already happened, and with the middle/index finger if the event is yet to happen.’ To ensure that participants understood the instructions, the experimenter repeated them, providing examples. In the past- and future-self projection conditions, for example, the experimenter encouraged participants to ‘project’ themselves in time, imagining to be 10 years back in the past/ahead in the future, focusing on their age 10 years ago/in 10 years and on the exact year it was/will be 10 years ago/in 10 years. Thus, while testing a 60-year-old participant in 2018, the experimenter would ask to imagine being 10 years in the past/future, when she was/will be 50/70, in 2008/2028. Participants were asked to describe the instructions back to the experimenter, and the instructions were repeated if the participant appeared to have misinterpreted them. In addition, before the experimental task, participants performed a brief practice session involving eight stimuli. The E-Prime 2.0 software was used for stimulus presentation and response collection. We recorded error rates and response times (RTs).

At the end of the experiment, to make sure that relative past/future responses to personal events were correct with respect to individual participants’ own life story and its chronology, all participants were asked, for each event presented in the past, present and future self-projection condition, to provide minimal information on the event, and to indicate whether the event was located in the past or the future with respect to the assumed time perspective. In the case of brain-damaged patients, the correctness of information provided during this interview was verified by consulting with the clinical staff or patients’ relatives. In the context of the interview, an event originally designed as relative past/future might turn out to belong to the other time category for a specific participant. For example, the event ‘driving licence’ features among the relative-past events (in the past self-projection condition) because most people in Italy obtain a driving license when they are about 20. However, if a participant obtained the driving license at 60, then for that participant the ‘driving licence’ event was included among the relative-future events, and a relative-future response was considered correct. These subject-specific adjustments in the assignment of events to the relative-future/past categories (4.7% of trials) allowed tailoring the set of personal events to each individual participant. During the interview, a participant might also note that a personal event was not relevant to their autobiography (e.g. they never got a car license and did not plan to), or that they did not know about a non-personal world event, in which case the item was removed from the dataset/analysis for that participant (0.01% of trials).

### Statistical analyses

Response times (i.e. for correct responses; RTs) were calculated by subtracting the duration of the acoustic presentation of the event from the overall response latency (as in Anelli *et al.*, 2016a,b). Response times (RTs) more/less than two standard deviations from each participant’s mean (10%) were excluded from the analysis (as in Anelli *et al.*, 2016a,b), as were data from two trials in which a vmPFC patient proved to not be familiar with the event (Gaddafi’s death). Error rates and RTs were entered in repeated-measures ANOVAs with *Group* (vmPFC patients, control patients and healthy controls) as between-subject factor, and *Event* (personal and non-personal), *Self-projection* (past, present and future) and *Self-reference* (relative-past event and relative-future event) as within-subject factors. *Post hoc* analyses were conducted with the Unequal N HSD test. To provide as informative an analysis of participants’ performance as possible, the relevant group differences were corroborated with non-parametric tests (see Supplementary Data) and individual modified single-subject *t*-tests (Crawford and Garthwaite, 2002). We report results significant at *P* < 0.05, two-tailed and η^2^_p_ as a measure of effect size.

## Results

### Error rates

**Fig. 3.**
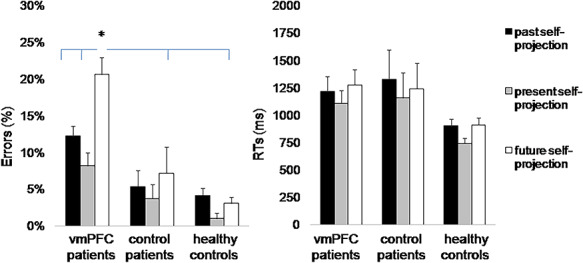
Self-projection. Mean error rates (A) and RTs (B) by participant group and self-projection (to the past, present and future time). Error bars depict standard errors of the mean. The asterisk indicates a significant difference (*P* < 0.05). We report RTs data to allow a more complete evaluation of performance in the different self-projection conditions, even though no group difference emerged for RTs.

The ANOVA on error rates revealed a significant effect of *Group* [F_(2,27)_ = 19.71, *P* < 0.0001, η^2^_p_ = 0.59] and a significant effect of *Self-projection* [F_(2,54)_ = 15.05, *P* < 0.001, η^2^_p_ = 0.36], qualified by a significant *Group* × *Self-projection* interaction [F_(4,54)_ = 5.96, *P* < 0.001, η^2^_p_ = 0.31] (Figure 3A). *Post hoc* comparisons showed that vmPFC patients made more errors in recognizing events while projected to the future (21%) compared to the past (12%, *P* = 0.004) and present (8%, *P* < 0.001), with no differences between the past and present self-projection conditions (*P* = 0.56). In contrast, in control patients (past self-projection = 5%, present self-projection = 4% and future self-projection = 7%) and healthy controls (past self-projection = 4%, present self-projection = 1% and future self-projection = 3%), errors were equally frequent across self-projection conditions (all *P*-values > 0.56). As a result, vmPFC patients made more errors in recognizing events than control patients (*P* = 0.018) and healthy controls (*P* = 0.001) in the future self-projection condition, but not in the past and present conditions (all *P*-values > 0.37), indicating a selective impairment with self-projection to the future in vmPFC patients. Control patients and healthy controls were equally accurate across conditions (all *P*-values > 0.95).

**Fig. 4.**
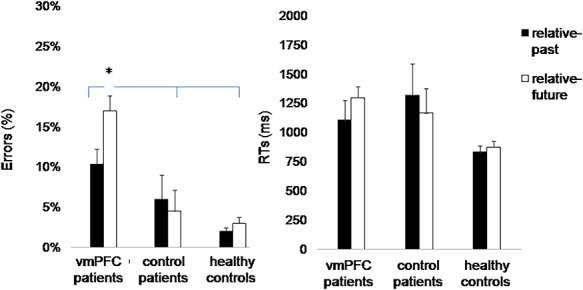
Self-reference. Mean error rates (A) and RTs (B) by participant group and self-reference (relative-past and relative-future events). Error bars depict standard errors of the mean. The asterisk indicates a significant difference (*P* < 0.05). We report RTs data to allow a more complete evaluation of performance with relative past *vs* future events, even though no group difference emerged for RTs.

There was also an effect of *Self-reference* [F_(1,27)_ = 4.26, *P* = 0.049, η^2^_p_ = 0.14], qualified by a significant interaction *Group* × *Self-reference* [F_(2,27)_ = 4.31, *P* = 0.02, η^2^_p_ = 0.24] (Figure 4A): vmPFC patients made more errors in recognizing relative-future compared to relative-past events (17% *vs* 10%, *P* = 0.027), whereas control patients (5% *vs* 6%, *P* = 0.98) and healthy controls (3% *vs* 2%, *P* = 0.96) were similarly accurate with relative-future and relative-past events. As a result, vmPFC patients misclassified more relative-future events than control patients (*P* = 0.003) and healthy controls (*P* < 0.001), but were equally accurate than the control groups with relative-past events (all *P*-values > 0.09), indicating a selective impairment in self-referencing future events in vmPFC patients. Control patients and healthy controls were equally accurate in classifying both relative-past and relative-future events (both *P*-values > 0.83).

We also found a significant *Event* × *Self-projection* interaction [F_(2,54)_ = 12.48, *P* < 0.0001, η^2^_p_ = 0.32], such that in the past self-projection condition (3% vs 10%, *P* < 0.001), but not in the future and present conditions (*P* > 0.50 in both cases), participants made fewer errors while evaluating personal compared to non-personal events, presumably reflecting participants’ better memory for their own past compared to non-personal events, especially when relatively remote events are involved (past self-projection condition). There was no other significant effect (*P* > 0.09 in all cases).

### Response times

The ANOVA on RTs revealed a significant main effect of *Group* [F_(2,27)_ = 3.83, *P* = 0.034, η^2^_p_ = 0.22] indicating generally slower RTs in vmPFC (1201 ms) and control patients (1243 ms) compared to healthy controls (853 ms). The main effect of *Self-projection* [F_(2,54)_ = 6.32, *P* = 0.003, η^2^_p_ = 0.19] was significant, such that all participants were slower at recognizing events while projected to the past (1074 ms) and to the future (1075 ms) than to the present (925 ms, both *P* = 0.003) (Figure 3B), replicating previous findings (Arzy *et al.*, 2008; Anelli *et al.*, 2018). The interaction *Event* × *Self-projection* was also significant [F_(2,54)_ = 4.31, *P* = 0.02, η^2^_p_ = 0.14], such that in the past self-projection condition (1159 *vs* 990 ms, *P* = 0.003), but not in the other conditions (*P* > 0.64 in both cases), participants were faster at recognizing personal compared to non-personal events, reflecting, again, better memory for the personal compared to the non-personal (remote) past.

Finally, there was a significant effect of *Self-reference* [F_(1,27)_ = 9.06, *P* = 0.006, η^2^_p_ = 0.25], qualified by a *Self-projection* × *Self-reference* interaction [F_(2,54)_ = 9.61, *P* < 0.001, η^2^_p_ = 0.26], indicating that in the present self-projection condition participants were faster at recognizing relative-past compared to relative-future events (804 *vs* 1088 ms, *P* < 0.001) (Figure 4B). This difference did not emerge in the past- and future self-projection conditions (both *P*-values > 0.93), which were characterized by generally longer RTs. There was no other significant effect (*P* > 0.06 in all cases).

### Individual patient’s behavior

The previous analyses revealed that vmPFC patients were impaired in two aspects of future thinking: projecting themselves to a future time perspective and evaluating future events. To verify whether these impairments were indeed registered in most of our vmPFC patients, we ran individual subject analyses comparing each patient’s performance to that of healthy controls with the Crawford’s modified *t*-test (Crawford and Garthwaite, 2002). We found a significant impairment in future self-projection (collapsing error rates across relative-future and relative-past personal and non-personal events) in all seven vmPFC patients (*t* > 2.77; *P* < 0.007, one-tailed). In contrast, this impairment was detected only in three of the seven control patients (*t* > 1.93; *P* < 0.03, one-tailed). This difference is significant (χ^2^ = 5.60, *P* = 0.02). Turning to self-reference, a significant impairment in recognizing relative-future events (collapsing error rates across self-projection conditions and type of event) was again detected in all seven vmPFC patients (*t* > 2.72; *P* < 0.008, one-tailed), but only in two of the seven control patients (*t* > 1.89; *P* < 0.04, one-tailed). This difference is significant (χ^2^ = 7.78, *P* = 0.005).

## Discussion

The present study investigated the effect of vmPFC damage on two component processes of MTT: the ability to project the self in time to assume different temporal perspectives (self-projection), and to determine, for each event in a series, whether it has already happened or is yet to happen relative to the currently assumed time perspective (self-reference). We found a striking asymmetry in the effect of vmPFC damage on both aspects of MTT, which hindered vmPFC patients’ possibility to project the self to the future, but not the past or the present, and to recognize relative-future but not relative-past events.

Before discussing, in turn, each aspect of vmPFC patients’ deficit in future-oriented MTT, we wish to emphasize that this deficit is not a common consequence of brain damage, for example reflective of a shortening of future time perspective following illness and perceived vulnerability (Ciaramelli *et al.*, 2019). Indeed, problems with future-oriented MTT were consistently present in vmPFC patients but not in control patients with brain damage not involving vmPFC. Our results are also unlikely to reflect poor comprehension of self-projection or task instructions on the vmPFC patients’ part. Indeed, all participants, including vmPFC patients, were slower at recognizing events while assuming a past or future (compared to present) time perspective, a robust finding reflecting the cognitive cost of self-projection (Arzy *et al.*, 2008, 2009; Anelli *et al.*, 2016a,b; Gauthier *et al.*, 2019). That this pattern was observed also in vmPFC patients strongly suggests they did indeed try and abandon the present moment to mentally move toward the subjective past and future. The vmPFC patients’ self-projection toward the future, however, went often awry, as indicated by an abnormal number of errors while processing events from that time perspective, as if patients failed at assuming (or maintaining) a future self-location, while they were normally capable to project the self back to the past. This finding aligns with recent neuroimaging evidence that during MTT the medial prefrontal cortex exhibit graded, progressively increasing activation with the succession of past, present and future self-projection (Gauthier *et al.*, 2019).

The selective deficit in future-oriented self-projection we detected in vmPFC patients stands in contrast to previous studies showing a pervasive impairment in remembering the past and imagining the future in vmPFC patients (Bertossi *et al.*, 2016a,b), which also characterizes amnesic patients with medial temporal lobe lesions (Race *et al.*, 2011). These findings, however, are only apparently in conflict. Indeed, previous studies had used MTT tasks requiring both the adoption of past and future temporal perspective and the construction of complex events from those perspectives (Bertossi *et al.*, 2016b), while the task we used here only involves the former. Thus, while impaired construction of both past and future experiences in previous studies may reflect a general problem in assembling detail-rich events, apparent even when vmPFC (as well as medial temporal lobe) patients have to construct atemporal experiences (Hassabis *et al.*, 2007; Bertossi *et al.*, 2016a; see also De Luca *et al.*, 2018), the present findings insulate an additional problem vmPFC patients have in assuming a future temporal perspective, above and beyond their event construction deficit, which affects MTT upstream. In contrast, patients with medial temporal lobe lesions have proven able to project the self in (future) time (Dalla Barba, 2001; Arzy *et al.*, 2009; Kwan *et al.*, 2013; Craver *et al.*, 2014), and their problems in imagining future experiences are likely to be fully explained by impaired event construction (McCormick *et al.*, 2019). Indeed, a single-case study of an amnesic patient with bilateral medial temporal damage using the same paradigm we have used here found no impairment in self-projection (Arzy *et al.*, 2009). Our findings make contact with previous evidence that vmPFC patients have more problems in constructing future compared to atemporal experiences (Bertossi *et al.*, 2016a), an asymmetry not present in medial temporal lobe amnesia (Hassabis  *et al.*, 2007), and that, when asked to enumerate personal future life events, vmPFC patients produce events less far ahead into the future than do brain-damaged controls, suggestive of a short future time perspective ([Bibr R35][Bibr R35]). Also, vmPFC patients (but, again, not medial temporal lobe amnesiacs; Kwan *et al.*, 2013) show increased delay discounting (Sellitto *et al.*, 2010; Peters and D’Esposito, 2016), in line with the view that they fail to conceive the future, even in purely semantic, abstract terms, hence devalue future rewards.

Why would vmPFC be necessary for future-oriented self-projection? It has been shown that self-projection to one’s personal past/future typically originates from the activation of high-level knowledge structures, such as schematic representations of life time periods and the self (e.g. when I graduated and when I will have a child), to then possibly converge on specific events of one’s personal timeline (Conway and Pleydell-Pearce, 2000; D’Argembeau and Mathy, 2011; D’Argembeau, 2020). Imagining the future relies more on schema-based knowledge than remembering the past, because we have no direct experience of future events (Anderson and Dewhurst, 2009; Berntsen and Bohr, 2010; Rubin, 2014). Knowledge about personal goals (e.g. I want to become a researcher) is especially effective in driving the construction of ones’ personal future (D’Argembeau and Mathy, 2011). The vmPFC is critical for appropriate processing of schema-related information (Burgess and Shallice, 1996; Ghosh *et al.*, 2014), including knowledge about the self (Philippi *et al.*, 2012; Verfaellie *et al.*, 2019) and personal goals (D’Argembeau *et al.*, 2010). We argue, therefore, that vmPFC patients failed to project the self to the future due to an inability in activating schematic knowledge critical to construct a mental representation of their personal future, so as to assume the perspective of their future self. This interpretation aligns with current views of the dynamics of MTT, according to which vmPFC initiates the activation of high-order autobiographical knowledge (e.g. lifetime periods and self schema), from which the hippocampus may then access (McCormick *et al.*, 2019; D’Argembeau, 2020; Dafni-Merom and Arzy, 2020; see also Barry *et al.*, 2019) or process (Schurr *et al.*, 2018) specific experiences.

Orthogonal to their impairment in future self-projection, vmPFC patients additionally showed a deficit in self-referencing future events, that is, in recognizing events lying ahead in the future with respect to their assumed location in subjective time (whether past, present or future), which were misclassified more often than relative-past events. Healthy as well as brain-damaged controls showed a comparable performance in recognizing relative-future and relative-past events, hence the selective deficit evinced by vmPFC patients with relative-future events is unlikely to merely reflect task difficulty. Note, also, that vmPFC patients’ false recognition of future events also emerged in the past self-location condition, that is, when dealing with events that were not actually future (with respect to the present time), and therefore it does not simply denote a problem in distinguishing familiar from novel events, or factual from potential, hypothetical events (see also Anelli *et al.*, 2018).

One is tempted to interpret vmPFC patients’ deficit as reflecting disordered chronology. The vmPFC, together with the basal forebrain, is indeed thought to support the correct assignment of memories to their correct place in time (Moscovitch, 1995; Tranel and Jones, 2006), and confabulation, a consequence of vmPFC damage, consists of memories that are often false in temporal context (Schnider, 2003; Gilboa *et al.*, 2006; Bertossi *et al.*, 2016b). Moreover, the vmPFC is engaged while individuals orient themselves in time (Peer *et al.*, 2015), and determining the temporal distance between the self and an event engages the prefrontal cortex (Gauthier *et al.*, 2019). Yet impaired chronology is again too general an interpretation for vmPFC patients’ performance in this task, as it would have led to an equal distribution of wrong attributions of relative-future events to the past and of relative-past events to the future, while vmPFC patients only showed an increased tendency to falsely recognize relative-future events as past.

According to one prominent view, the timing of one’s memories is not indicated by stable ‘time tags’ marking each of them in succession (Friedman, 1993), but dynamically reconstructed by strategic processes depending on retrieval goals (Burgess and Shallice, 1996). We propose that vmPFC patients’ false recognition of relative-future events reveals a specific deficit in monitoring novelty signals originating from events that, with respect to the current self-position in time, are yet to happen, which felt wrongly familiar. This deficit is reminiscent of other instances of false recognition in vmPFC patients. For example, in recognition memory tasks, vmPFC patients falsely recognize distractors that were targets in previous runs of the experiment (Schnider, 2003), or with which they had pre-experimental experience (Ciaramelli and Ghetti, 2007), as if they failed to appreciate that, in the context of the current run or experiment, they were novel. More generally, vmPFC patients tend to assimilate irrelevant information into activated schemata (Ghosh *et al.*, 2014). One could argue, therefore, that vmPFC patients failed at using a schema of the current reality to identify (future) events that mismatched the schema because they were not previously experienced. This deficit had an impact on recognition of personal as well as general relative-future events, consistent with previous evidence of false recognition in both domains following vmPFC damage (Schnider, 2003; Gilboa *et al.*, 2006; Ciaramelli and Ghetti, 2007), in fact depriving vmPFC patients fully of a view of the future.

We end by noting some limitations and future directions of our work. The sample size is small, and therefore some effects may have gone undetected due to limited statistical power. Future studies involving more vmPFC patients will help confirm the selective deficit we observed in future MTT and link it to specific vmPFC subregions. Also, our results show that, even though control patients did not show a future MTT impairment at the group level, a few of them did, suggesting that other (e.g. temporo-parietal) regions may be causally linked to future self-projection and self-reference. Again, testing this hypothesis will require recruiting larger samples of patients, and with more homogeneous lesion sites than our control patient group. Finally, the task we used can detect whether patients place events correctly in the (relative) past and future, but not whether they would put the events in the correct chronological order with respect to one another within the past or the future. Thus, a future direction of the study is to test whether the disadvantage observed in vmPFC patients in processing future events is limited to the recognition of events, or it would extend to their ordering.

In summary, we have shown that vmPFC patients have a multifaceted impairment of future MTT, being unable to project themselves to the future, and to anticipate the events that await them in the future, pointing to a prominent role of vmPFC in future-oriented cognition. Future self-projection and self-reference have a profound impact on future-oriented choice: taking the perspective of one’s future self reduces discounting of future rewards (Peters and Büchel, 2010), and the anticipation of future events is associated with goal-directed behavior and motivation (Boyer, 2008). Thus, the future-oriented MTT deficit we detected in vmPFC patients is likely to play a role in their steep delay discounting (Sellitto *et al.*, 2010), poor problem-solving (Peters *et al.*, 2017) and apathy (Fellows and Farah, 2005). If so, cueing future thinking (Peters and Büchel, 2010), or the deployment of spatial attention toward future locations of the mental timeline (Anelli *et al.*, 2016b), may be effective in pushing patients’ temporal horizon ahead into the future, reducing their shortsightedness.

## Supplementary Material

nsaa163_Supp

## References

[R1] Addis D.R. , Wong A.T., Schacter D.L. (2007). Remembering the past and imagining the future: common and distinct neural substrates during event construction and elaboration. *Neuropsychologia*, 45, 1363–77. 17126370 10.1016/j.neuropsychologia.2006.10.016 PMC1894691

[R2] Anelli F. , Ciaramelli E., Arzy S., Frassinetti F. (2016a). Age-related effects on future mental time travel. *Neural Plasticity*, 1867270. 10.1155/2016/1867270 PMC4838805 27144031

[R3] Anelli F. , Ciaramelli E., Arzy S., Frassinetti F. (2016b). Prisms to travel in time: investigation of time-space association through prismatic adaptation effect on mental time travel. *Cognition*, 156, 1–5. 27467891 10.1016/j.cognition.2016.07.009

[R4] Anelli F. , Peters-Founshtein G., Shreibman Y., et al. (2018). Nature and nurture effects on the spatiality of the mental time line. *Scientific Reports*, 8, 11710. 10.1038/s41598-018-29584-3 PMC6076263 30076378

[R5] Anderson R.J. , Dewhurst S.A. (2009). Remembering the past and imagining the future: differences in event specificity of spontaneously generated thought. *Memory*, 17, 367–73. 19235018 10.1080/09658210902751669

[R6] Arzy S. , Bick A., Blanke O. (2009). Mental time in amnesia: evidence from bilateral medial temporal damage before and after recovery. *Cognitive Neuropsychology*, 26, 503–10. 20029695 10.1080/02643290903439178

[R7] Arzy S. , Molnar-Szakacs I., Blanke O. (2008). Self in time: imagined self-location influences neural activity related to mental time travel. *Journal of Neuroscience*, 25, 6502–7. 10.1523/JNEUROSCI.5712-07.2008 PMC6670885 18562621

[R8] Atance C.M. , O’Neill D.K. (2001). Episodic future thinking. *Trends in Cognitive Sciences*, 5, 533–9. 11728911 10.1016/s1364-6613(00)01804-0

[R9] Baird B. , Smallwood J., Schooler J.W. (2011). Back to the future: autobiographical planning and the functionality of mindwandering. *Consciousness and Cognition*, 20, 1604–11. 21917482 10.1016/j.concog.2011.08.007

[R10] Barry D.N. , Barnes G.R., Clark I.A., Maguire E.A. (2019). The neural dynamics of novel scene imagery. *Journal of Neuroscience*, 39, 4375–86. 30902867 10.1523/JNEUROSCI.2497-18.2019 PMC6538850

[R11] Bechara A. , Damasio A.R., Damasio H., Anderson S.W. (1994). Insensitivity to future consequences following damage to human prefrontal cortex. *Cognition*, 50, 7–15. 8039375 10.1016/0010-0277(94)90018-3

[R12] Bertossi E. , Aleo F., Braghittoni D., Ciaramelli E. (2016a). Stuck in the here and now: construction of fictitious and future experiences following ventromedial prefrontal damage. *Neuropsychologia*, 81, 107–16. 26707714 10.1016/j.neuropsychologia.2015.12.015

[R13] Bertossi E. , Candela V., De Luca F., Ciaramelli E. (2017). Episodic future thinking following vmPFC damage: impaired event construction, maintenance, or narration? *Neuropsychology*, 31, 337–48. 28054822 10.1037/neu0000345

[R14] Bertossi E. , Ciaramelli E. (2016). Ventromedial prefrontal damage reduces mind-wandering and biases its temporal focus. *Social Cognitive and Affective Neuroscience*, 11, 1783–91. 27445210 10.1093/scan/nsw099 PMC5091689

[R15] Bertossi E. , Tesini C., Cappelli A., Ciaramelli E. (2016b). Ventromedial prefrontal cortex damage causes a pervasive impairment in episodic remembering and future thinking. *Neuropsychologia*, 90, 12–24. 26827916 10.1016/j.neuropsychologia.2016.01.034

[R16] Berntsen D. , Bohn A. (2010). Remembering and forecasting: the relation between autobiographical memory and episodic future thinking. *Memory & Cognition*, 38, 265–78. 20234017 10.3758/MC.38.3.265

[R17] Berryhill M. , Picasso L., Arnold R., Drowos D., Olson I. (2010). Similarities and differences between parietal and frontal patients in autobiographical and constructed experience tasks. *Neuropsychologia*, 48, 1385–93. 20096710 10.1016/j.neuropsychologia.2010.01.004 PMC2843776

[R18] Boyer P. (2008). Evolutionary economics of mental time travel? *Trends in Cognitive Sciences*, 12, 219–24. 18468941 10.1016/j.tics.2008.03.003

[R19] Buckner R.L. , Carroll D.C. (2007). Self-projection and the brain. *Trends in Cognitive Sciences*, 11, 49–57. 17188554 10.1016/j.tics.2006.11.004

[R20] Burgess P.W. , Shallice T. (1996). Confabulation and the control of recollection. *Memory*, 4, 359–411. 8817460 10.1080/096582196388906

[R21] Caruso E. , Gilbert D.T., Wilson T.D. (2008). A wrinkle in time: asymmetric valuation of past and future events. *Psychological Science*, 19, 796–801. 18816287 10.1111/j.1467-9280.2008.02159.x

[R22] Ciaramelli E. , Ghetti S. (2007). What are confabulators’ memories made of? A study of subjective and objective measures of recollection in confabulation. *Neuropsychologia*, 45, 1489–500. 17222872 10.1016/j.neuropsychologia.2006.11.007

[R23] Ciaramelli E. , Giannetti C., Orsini R. (2019). Does death make us all equal? Materialism and status-seeking under mortality salience. *International Review of Economics*, 66, 57–78.

[R24] Conway M.A. , Pleydell-Pearce C.W. (2000). The construction of autobiographical memories in the self-memory system. *Psychological Review*, 107, 261–88. 10789197 10.1037/0033-295x.107.2.261

[R25] Craver C.F. , Kwan D., Steindam C., Rosenbaum R.S. (2014). Individuals with episodic amnesia are not stuck in time. *Neuropsychologia*, 57, 191–5. 24680757 10.1016/j.neuropsychologia.2014.03.004

[R26] Crawford J.R. , Garthwaite P.H. (2002). Investigation of the single case in neuropsychology: confidence limits on the abnormality of test scores and test score differences. *Neuropsychologia*, 40, 1196–208. 11931923 10.1016/s0028-3932(01)00224-x

[R27] Dafni-Merom A. , Arzy S. (2020). The radiation of autonoetic consciousness in cognitive neuroscience: a functional neuroanatomy perspective. *Neuropsychologia*, 143, 107477. 10.1016/j.neuropsychologia.2020.107477 32360475

[R28] D’Argembeau A. (2020). Zooming in and out on one’s life: autobiographical representations at multiple time scales. *Journal of Cognitive Neuroscience*, 32, 2037–55. 32163320 10.1162/jocn_a_01556

[R29] D’Argembeau A. , Mathy A. (2011). Tracking the construction of episodic future thoughts. *Journal of Experimental Psychology: General*, 140, 258–71. 21401291 10.1037/a0022581

[R30] D’Argembeau A. , Stawarczyk D., Majerus S., et al. (2010). The neural basis of personal goal processing when envisioning future events. *Journal of Cognitive Neuroscience*, 22, 1701–13. 19642887 10.1162/jocn.2009.21314

[R31] D’Argembeau A. , Van der Linden M. (2004). Phenomenal characteristics associated with projecting oneself back into the past and forward into the future: influence of valence and temporal distance. *Consciousness and Cognition*, 13, 844–58. 15522635 10.1016/j.concog.2004.07.007

[R32] De Luca F. , McCormick C., Mullally S.L., Intraub H., Maguire E.A., Ciaramelli E. (2018). Boundary extension is attenuated in patients with ventromedial prefrontal cortex damage. *Cortex*, 108, 1–12. 30086391 10.1016/j.cortex.2018.07.002 PMC6238077

[R33] de Vito S. , Gamboz N., Brandimonte M.A., Barone P., Amboni M., Della Sala S. (2012). Future thinking in Parkinson’s disease: an executive function? *Neuropsychologia*, 50, 1494–501. 22406693 10.1016/j.neuropsychologia.2012.03.001

[R34] Duval C. , Desgranges B., de La Sayette V., Belliard S., Eustache F., Piolino P. (2012). What happens to personal identity when semantic knowledge degrades? A study of the self and autobiographical memory in semantic dementia. *Neuropsychologia*, 50, 254–65. 22155259 10.1016/j.neuropsychologia.2011.11.019

[R35] Fellows L.K. , Farah M.J. (2005). Dissociable elements of human foresight: a role for the ventromedial frontal lobes in framing the future, but not in discounting future rewards. *Neuropsychologia*, 43, 1214–21. 15817179 10.1016/j.neuropsychologia.2004.07.018

[R36] Friedman W.J. (1993). Memory for the time of past events. *Psychological Bulletin*, 113, 44–66.

[R37] Gauthier B. , Pestke K., van Wassenhove V. (2019). Building the arrow of time… over time: a sequence of brain activity mapping imagined events in time and space. *Cerebral Cortex*, 29, 4398–414. 30566689 10.1093/cercor/bhy320

[R38] Gauthier L. , Dehaut F., Joanette Y. (1989). The Bells test: a quantitative and qualitative test for visual neglect. *International Journal of Clinical Neuropsychology*, 11, 49–54.

[R39] Ghosh V.E. , Moscovitch M., Melo Colella B., Gilboa A. (2014). Schema representation in patients with ventromedial PFC lesions. *Journal of Neuroscience*, 34, 12057–70. 25186751 10.1523/JNEUROSCI.0740-14.2014 PMC6608465

[R40] Gilboa A. , Alain C., Stuss D.T., Melo B., Miller S., Moscovitch M. (2006). Mechanisms of spontaneous confabulations: a strategic retrieval account. *Brain*, 129, 1399–414. 16638795 10.1093/brain/awl093

[R41] Hassabis D. , Kumaran D., Vann S.D., Maguire E.A. (2007). Patients with hippocampal amnesia cannot imagine new experiences. *Proceedings of the National Academy of Sciences of the USA*, 104, 1726–31. 17229836 10.1073/pnas.0610561104 PMC1773058

[R42] Irish M. , Addis D.R., Hodges J.R., Piguet O. (2012). Considering the role of semantic memory in episodic future thinking: evidence from semantic dementia. *Brain*, 135, 2178–91. 22614246 10.1093/brain/aws119

[R43] Irish M. , Piguet O. (2013). The pivotal role of semantic memory in remembering the past and imagining the future. *Frontiers in Behavioral Neuroscience*, 7, 1–10. 23565081 10.3389/fnbeh.2013.00027 PMC3615221

[R44] Kurczek J. , Wechsler E., Ahuja S., et al. (2015). Differential contributions of hippocampus and medial prefrontal cortex to self-projection and self-referential processing. *Neuropsychologia*, 73, 116–26. 25959213 10.1016/j.neuropsychologia.2015.05.002 PMC4671497

[R45] Kwan D. , Craver C.F., Green L., Myerson J., Rosenbaum R.S. (2013). Dissociations in future thinking following hippocampal damage: evidence from discounting and time perspective in episodic amnesia. *Journal of Experimental Psychology: General*, 142, 1355–69. 23978187 10.1037/a0034001

[R46] Lieberman M.D. , Straccia M.A., Meyer M.L., Du M., Tan K.M. (2019). Social, self, (situational), and affective processes in medial prefrontal cortex (MPFC): causal, multivariate, and reverse inference evidence. *Neuroscience and Biobehavioral Reviews*, 99, 311–28. 30610911 10.1016/j.neubiorev.2018.12.021

[R47] McCormick C. , Ciaramelli E., De Luca F., Maguire E.A. (2018). Comparing and contrasting the effects of hippocampal and ventromedial prefrontal cortex damage: a review of human lesion studies. Neuroscience, 374, 295–318. 28827088 10.1016/j.neuroscience.2017.07.066 PMC6053620

[R48] Mancuso M. , Rosadoni S., Capitani D., et al. (2015). Italian standardization of the Apples Cancellation Test. *Neurological Sciences*, 36, 1233–40. 25618236 10.1007/s10072-015-2088-2

[R49] Moscovitch M. (1995). Confabulation. In: Schacter, D.L., editor. *Memory Distortions*. Cambridge: Harvard University Press, 226–51.

[R50] Nyberg L. , Kim A.S.N., Habib R., Levine B., Tulving E. (2010). Consciousness of subjective time in the brain. *Proceedings of the National Academy of Sciences of the USA*, 107, 22356–9. 21135219 10.1073/pnas.1016823108 PMC3009795

[R51] Okuda J. , Fujii T., Ohtake H., et al. (2003). Thinking of the future and past: the roles of the frontal pole and the medial temporal lobes. *NeuroImage*, 19, 1369–80. 12948695 10.1016/s1053-8119(03)00179-4

[R52] Peer M. , Salomon R., Goldberg I., Blanke O., Arzy S. (2015). Brain system for mental orientation in space, time, and person. *Proceedings of the National Academy of Sciences of the USA*, 112, 11072–7. 26283353 10.1073/pnas.1504242112 PMC4568229

[R53] Peters J. , Büchel C. (2010). Episodic future thinking reduces reward delay discounting through prefrontal–mediotemporal interactions. *Neuron*, 66, 138–48. 20399735 10.1016/j.neuron.2010.03.026

[R54] Peters J. , D’Esposito M. (2016). Effects of medial orbitofrontal cortex lesions on self-control in intertemporal choice. *Current Biology*, 26, 2625–8. 27593380 10.1016/j.cub.2016.07.035

[R55] Peters S.L. , Fellows L.K., Sheldon S. (2017). The ventromedial frontal lobe contributes to forming effective solutions to real-world problems. *Journal of Cognitive Neuroscience*, 29, 991–1001. 27991183 10.1162/jocn_a_01088

[R56] Philippi C.L. , Duff M.C., Denburg N.L., Tranel D., Rudrauf D. (2012). Medial PFC damage abolishes the self-reference effect. *Journal of Cognitive Neuroscience*, 24, 475–81. 21942762 10.1162/jocn_a_00138 PMC3297026

[R57] Race E. , Keane M.M., Verfaellie M. (2011). Medial temporal lobe damage causes deficits in episodic memory and episodic future thinking not attributable to deficits in narrative construction. *Journal of Neuroscience*, 31, 10262–9. 21753003 10.1523/JNEUROSCI.1145-11.2011 PMC4539132

[R58] Rasmussen K.W. , Berntsen D. (2018). Deficits in remembering the past and imagining the future in patients with prefrontal lesions. *Journal of Neuropsychology*, 12, 78–100. 27339103 10.1111/jnp.12108

[R59] Rogers T.B. , Kuiper N.A., Kirker W.S. (1977). Self-reference and the encoding of personal information. *Journal of Personality and Social Psychology*, 35, 677–88. 909043 10.1037//0022-3514.35.9.677

[R60] Romero K. , Moscovitch M. (2012). Episodic memory and event construction in aging and amnesia. *Journal of Memory and Language*, 67, 270–84.

[R61] Rorden C. , Brett M. (2000). Stereotaxic display of brain lesions. *Behavioural Neurology*, 12, 191–200. 11568431 10.1155/2000/421719

[R62] Rosenbaum R.S. , Gilboa A., Levine B., Winocur G., Moscovitch M. (2009). Amnesia as an impairment of detail generation and binding: evidence from personal, fictional, and semantic narratives in K.C. *Neuropsychologia*, 47, 2181–7. 19100757 10.1016/j.neuropsychologia.2008.11.028

[R63] Rubin D.C. (2014). Schema-driven construction of future autobiographical traumatic events: the future is much more troubling than the past. *Journal of Experimental Psychology: General*, 143, 612–30. 23607632 10.1037/a0032638 PMC3778053

[R64] Rubin D.C. , Rahhal T.A., Poon L.W. (1998). Things learned in early adulthood are remembered best. *Memory & Cognition*, 26, 3–19. 9519693 10.3758/bf03211366

[R65] Schacter D.L. , Addis D.R., Hassabis D., Martin V.C., Spreng R.N., Szpunar K.K. (2012). The future of memory: remembering, imagining, and the brain. *Neuron*, 76, 677–94. 23177955 10.1016/j.neuron.2012.11.001 PMC3815616

[R66] Schnider A. (2003). Spontaneous confabulation and the adaptation of thought to ongoing reality. *Nature Reviews Neuroscience*, 4, 662–71. 12894241 10.1038/nrn1179

[R67] Schurr R. , Nitzan M., Eliahou R., et al. (2018). Temporal dissociation of neocortical and hippocampal contributions to mental time travel using intracranial recordings in humans. *Frontiers in Computational Neuroscience*, 12, 1–12. 29541024 10.3389/fncom.2018.00011 PMC5835533

[R68] Sellitto M. , Ciaramelli E., Di Pellegrino G. (2010). Myopic discounting of future rewards after medial orbitofrontal damage in humans. *Journal of Neuroscience*, 30, 16429–36. 21147982 10.1523/JNEUROSCI.2516-10.2010 PMC6634874

[R69] Spinnler H. , Tognoni G. (1987). Standardizzazione e taratura Italiana di test neuropsicologici. *Italian Journal of Neurological Sciences*, 8, 71–95. 3330072

[R70] Stawarczyk D. , Cassol H., D’Argembeau A. (2013). Phenomenology of future-oriented mind-wandering episodes. *Frontiers in Psychology*, 4, 425. 10.3389/fpsyg.2013.00425 PMC3712143 23882236

[R71] Suddendorf T. , Corballis M.C. (2007). The evolution of foresight: what is mental time travel, and is it unique to humans? *Behavioural and Brain Sciences*, 30, 299–351. 10.1017/S0140525X07001975 17963565

[R72] Tranel D. , Jones R.D. (2006). Knowing ‘what’ and knowing ‘when’. *Journal of Clinical and Experimental Neuropsychology*, 28, 43–66. 16448975 10.1080/13803390490919344

[R73] Tulving E. (2002). In: Stuss, D.T., Knight, R.C., editors. *Principles of Frontal Lobe Functions*. *Chronesthesia: Conscious awareness of subjective time*. New York: Oxford University Press, 311–25.

[R74] Verfaellie M. , Wank A.A., Reid A.G., Race E., Keane M.M. (2019). Self-related processing and future thinking: distinct contributions of ventromedial prefrontal cortex and the medial temporal lobes. *Cortex*, 115, 159–71. 30826623 10.1016/j.cortex.2019.01.028 PMC6513722

[R75] Weiler J.A. , Suchan B., Koch B., Schwarz M., Daum I. (2011). Differential impairments of remembering the past and imagining novel events after thalamic lesions. *Journal of Cognitive Neuroscience*, 23, 3037–51. 21268672 10.1162/jocn.2011.21633

[R76] Wheeler M.A. , Stuss D.T., Tulving E. (1997). Toward a theory of episodic memory: the frontal lobe and autonoetic conscious ness. *Psychonomic Bulletin*, 121, 331–54. 10.1037/0033-2909.121.3.331 9136640

