# Supplementary material for: An asymmetry in past and future mental time travel following vmPFC damage
Source: Soc Cogn Affect Neurosci. 2020 Dec 31;16(3):315–25. doi: 10.1093/scan/nsaa163 (PMC7943363; doi:10.1093/scan/nsaa163)
Supplement: nsaa163_Supp [file nsaa163_supp.zip › Supplementary.docx]

Supplementary material for:

**An asymmetry in past and future mental time travel following vmPFC damage**

Elisa Ciaramelli^1,2^, Filomena Anelli^1,3^, Francesca Frassinetti^1,4^

^1^Department of Psychology, University of Bologna, viale C. Berti-Pichat 5, Bologna, Italy

^2^Centro studi e ricerche in Neuroscienze Cognitive, via Rasi e Spinelli 176, Cesena, Italy

^3^currently at: Department of Surgical and Medical Sciences, "Magna Graecia" University of Catanzaro, viale Europa - Loc. Germaneto, Catanzaro, Italy

4.Istituti Clinici Scientifici Maugeri IRCCS, Operative Unit for Recovery and Functional Rehabilitation, Institute of Castel Goffredo, Mantova, Italy

**Supplementary Table.** List of personal and non-personal world events used.

|  | **PAST**  **SELF-PROJECTION** | **PRESENT**  **SELF- PROJECTION** | **FUTURE**  **SELF- PROJECTION** |
| --- | --- | --- | --- |
| **PERSONAL EVENTS (RELATIVE PAST)** | First boyfriend/girlfriend | Using glasses | Using glasses |
|  | First car | 40^th^ birthday | 80^th^ birthday |
|  | First salary | First child | Silver wedding |
|  | Driving licence | First car | First grandchild |
|  | 40^th^ birthday | Retirement | First trip by train |
|  | First day of school | First hospitalization | Golden wedding |
| **PERSONAL EVENTS (RELATIVE FUTURE)** | 80^th^ birthday | 80^th^ birthday | Living on the moon |
|  | Son retirement | First great grandchild | Son retirement |
|  | First great grandchild | Grandchild wedding | Diamond wedding |
|  | Grandchild wedding | Diamond wedding | Grandchild wedding |
|  | Admission to the nursing home | Admission to the nursing home | Flying to Mars |
|  | Golden wedding | Son retirement | 100^th^ birthday |
| **NON-PERSONAL EVENTS**  **(RELATIVE PAST)** | President Pertini’s election | Obama's election | Obama's election |
|  | Fall of Berlin wall | First use of euro | First use of euro |
|  | Chernobyl disaster | Chernobyl disaster | Europe unites |
|  | Man on the moon | Man on the moon | Gaddafi’s death |
|  | September 11^th^  Earthquake in Irpinia | September 11^th^  Rita Levi Montalcini’s death | Pope Francesco’s election  Ratzinger’s retirement |
| **NON-PERSONAL EVENTS**  **(RELATIVE FUTURE)** | Pope Francesco’s election | Peace in middle east | Flying car |
|  | Gaddafi’s death | Completely defeat illnesses | World peace |
|  | Woman president in USA | Woman president in USA | Completely defeat mafia |
|  | Rita Levi Montalcini’s death | Completely defeat mafia | Completely defeat illness |
|  | End of the world | End of the world | End of the world |
|  | Completely defeat mafia | Completely defeat world hunger | Completely defeat world hunger |

**Supplementary analyses**

To provide as informative an analysis of participants' performance as possible, the relevant group differences were confirmed using less powerful, but more robust, nonparametric tests. We analyzed comparisons involving the three participant groups with non-parametric Kruskall-Wallis analyses of variance (ANOVA) and conducted planned comparisons between vmPFC patients and brain-damaged and healthy controls with Mann-Whitney tests. We report results significant at p < 0.05, two-tailed, and η^2^_p_ as a measure of effect size.

We found group differences in recognizing relative-future events (collapsing across personal and non-personal events) across the future (H = 13.37, p = 0.001, η^2^_p_ = 0.42), past (H = 12.52, p = 0.002, η^2^_p_ = 0.39), and present self-projection conditions (H = 11.29; p = 0.003, η^2^_p_ = 0.34). Specifically, vmPFC patients made more errors in recognizing relative-future events than healthy (future condition: z = 3.67, p < .001, η^2^_p_ = 0.58; past condition: z = 3.17, p = 0.002, η^2^_p_ = 0.43; present condition: z = 2.70, p = 0.007, η^2^_p_ = 0.31) and brain-damaged controls (future condition: z = 1.98, p = 0.047, η^2^_p_ = 0.28; past condition: z = 2.55, p = 0.01, η^2^_p_ = 0.46; present condition: z = 2.04, p = 0.04, η^2^_p_ = 0.29), with no differences between the control groups (p > 0.41, η^2^_p_ < 0.04 in all cases). Group differences in recognizing relative-past events emerged, again, in the future-self-projection condition (H = 7.35, p = 0.03, η^2^_p_ = 0.20), with more errors in vmPFC patients than healthy controls (z = 2.59, p = 0.009, η^2^_p_ = 0.22), but not in the past (H = 2.46, p = 0.29, η^2^_p_ = 0.02) or present self-projection condition (H = 5.85, p = 0.053, η^2^_p_ = 0.14). Thus, the non-parametric analyses confirmed that vmPFC patients had more marked problems in recognizing relative-future events, and in recognizing events from a future self-perspective.

To directly quantify vmPFC patients' disadvantage with future self-reference and self-projection, we calculated a 'future self-reference index', by subtracting errors in recognizing relative-past events from those in recognizing relative-future events, and a 'future self-projection index', by subtracting errors made in the past and present self-projection conditions (collapsed) from those in the future self-projection condition. We found significant group differences in both the future self-reference index (H = 8.97, p = 0.01, η^2^_p_ = 0.26) and the future self-projection index (H = 12.21, p = 0.002, η^2^_p_ = 0.37): vmPFC patients had a higher future self-reference index than healthy controls (6.60% vs. 1.02%; z = 2.74; p = 0.006, η^2^_p_ = 0.32) and control patients (6.60% vs. -1.25%; z = 2.23, p = 0.02, η^2^_p_ = 0.35), and a higher self-projection index compared to healthy controls (10.43% vs. 0.27%; z = 3.61, p < 0.001, η^2^_p_ = 0.56) and, marginally, control patients (10.43% vs. 2.96%; z = 1.85, p = 0.06, η^2^_p_ = 0.24), confirming they were particularly disadvantaged in recognizing future events and in projecting the self to the future. There were no differences in the self-reference and self-projection indices between healthy and brain-damaged controls (p > 0.30, η^2^_p_ < 0.05 in both cases).
